# A neuroendocrine tumor within an anterior mediastinal mature teratoma: a case report

**DOI:** 10.1186/s13019-022-02091-3

**Published:** 2022-12-22

**Authors:** Daisuke Sato, Asami Izu, Masashi Sakakibara, Sohei Hayashi, Riken Kawachi, Mie Shimamura, Shinobu Masuda, Hiroyuki Sakurai

**Affiliations:** 1 Department of Respiratory Surgery, Nihon University School of Medicine, 30-1, Ohyaguchi-Kamicho, Itabashi-Ku, Tokyo, 173-8610 Japan; 2 Department of Pathology and Microbiology, Nihon University School of Medicine, 30-1, Ohyaguchi-Kamicho, Itabashi-Ku, Tokyo, 173-8610 Japan

**Keywords:** Germ cell tumor with somatic type malignancy, Malignant transformation, Neuroendocrine tumor, Mediastinum, Teratoma

## Abstract

**Background:**

Mature teratomas are benign germ cell tumors. On rare occasions, they have been associated with somatic malignancies and are termed rare germ cell tumors with a somatic-type malignancy (GCTSM). Mature teratomas commonly comprise adenocarcinomas; only seven previous cases of mature teratomas with neuroendocrine tumors have been reported to date. Here, we report a patient with a neuroendocrine tumor whithin a mature teratoma.

**Case presentation:**

A 26-year-old man visited our department complaining of chest tightness. Contrast-enhanced computed tomography (CT) scans showed a strongly enhanced lesion within a 10-cm encapsulated cystic lesion in the anterior mediastinum. Positron emission tomography (PET) scans showed no areas of significant ^18^F-fluorodeoxyglucose (^18^F-FDG) accumulation. He underwent complete tumor resection via the transsternal approach. Histopathological examination of the specimen indicated a neuroendocrine tumor contained within a mature teratoma.

**Conclusions:**

In this case, a neuroendocrine tumor was contained within a mature teratoma. Our patient had no specific symptoms and his serum markers were within the normal range. Although PET is beneficial for diagnosing other GCTSM, it is not useful in detecting a neuroendocrine tumor. Therefore, the preoperative diagnosis of neuroendocrine tumors contained within mature teratomas remains challenging. However, GCTSM should be suspected in patients exhibiting CT findings of a mediastinal tumor, measuring ≥ 6 cm, in addition to characteristic GCTSM findings. Moreover, surgery should be performed carefully in such cases.

## Background

Mature teratomas are the most common benign germ cell tumors arising in the mediastinum [1]. Mature teratomas rarely contain malignant components. Germ cell tumors accompanied with a somatic-type malignant component of sarcoma, carcinoma, or both, are defined as germ cell tumors with a somatic type malignancy (GCTSM), according to the World Health Organization’s most recent histologic classification [1]. Among the malignant tumors associated with GCTSM, sarcoma is the most frequently described malignancy, followed by adenocarcinoma and squamous cell carcinoma, whereas neuroendocrine tumors are rare [1]. Most GCTSM are difficult to diagnose preoperatively. Although previous reports have shown that PET is useful for the diagnosis of mature teratomas with adenocarcinoma or squamous cell carcinoma, no studies have used PET or reported its diagnostic role in relation to mature teratomas with a neuroendocrine tumor.

Herein, we report the case of a 26-year-old man with a neuroendocrine tumor contained within a mature teratoma and discuss the relevant literature.

## Case presentation

**Fig. 1:**
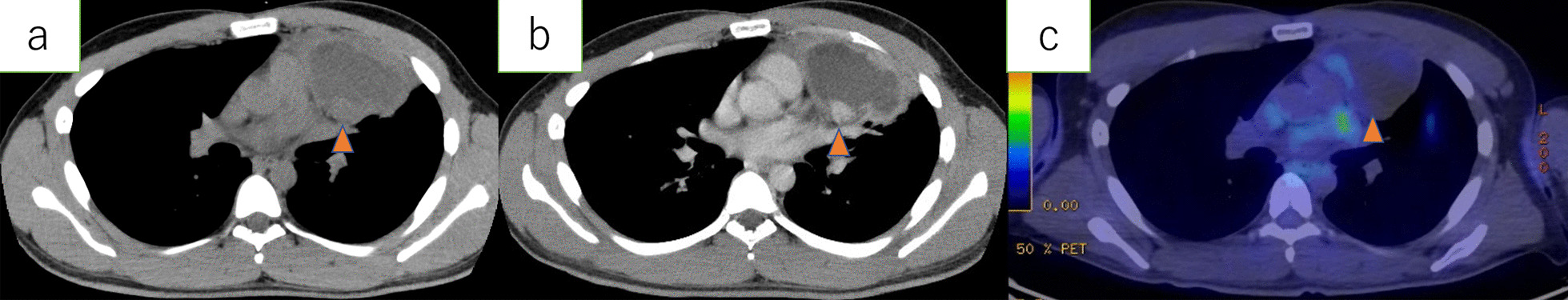
Chest computed tomography and positron-emission tomography showing the characteristics of the anterior mediastinal lesion. **a** Chest computed tomography scan shows a capsulated cystic lesion in the anterior mediastinum and a nodular lesion (arrow) on the capsule inside the cystic lesion wall. **b** The nodular lesion (arrow) exhibits strong enhancement after the intravenous administration of contrast material. **c** Positron-emission tomography shows no significant ^18^F-fluorodeoxyglucose accumulation in the nodular lesion (arrow)

A 26-year-old man visited our department complaining of chest tightness. He had no relevant medical or family history and was a non-smoker. His physical findings were essentially normal. His hematological, biochemical, coagulation, and serum tumor marker results were all within the normal range. Chest computed tomography (CT) scans showed a 10-cm encapsulated cystic lesion in the anterior mediastinum, along with a 1.5-cm nodular lesion on the capsule located inside the cystic lesion wall (Fig. 1a). The nodular lesion had clear boundaries and was strongly enhanced on CT after intravenous administration of the contrast material (Fig. 1b). Positron emission tomography (PET), which was performed 60 min after the injection of ^18^F-fluorodeoxyglucose (^18^F-FDG), showed no areas of significant ^18^F-FDG accumulation (Fig. 1c). Therefore, we preoperatively diagnosed him with a mature teratoma rather than a malignant mediastinal tumor or thymoma, based on the radiology findings. Surgery via the transsternal approach was then performed to diagnose and treat the lesion. The mediastinal lesion was located on the anterior mediastinum, proximal to the left pulmonary artery and left upper lobe. Complete resection of the mediastinal lesion and wedge resection of the anterior portion of the left upper lobe were performed based on the extent of the tumor.

**Fig. 2:**
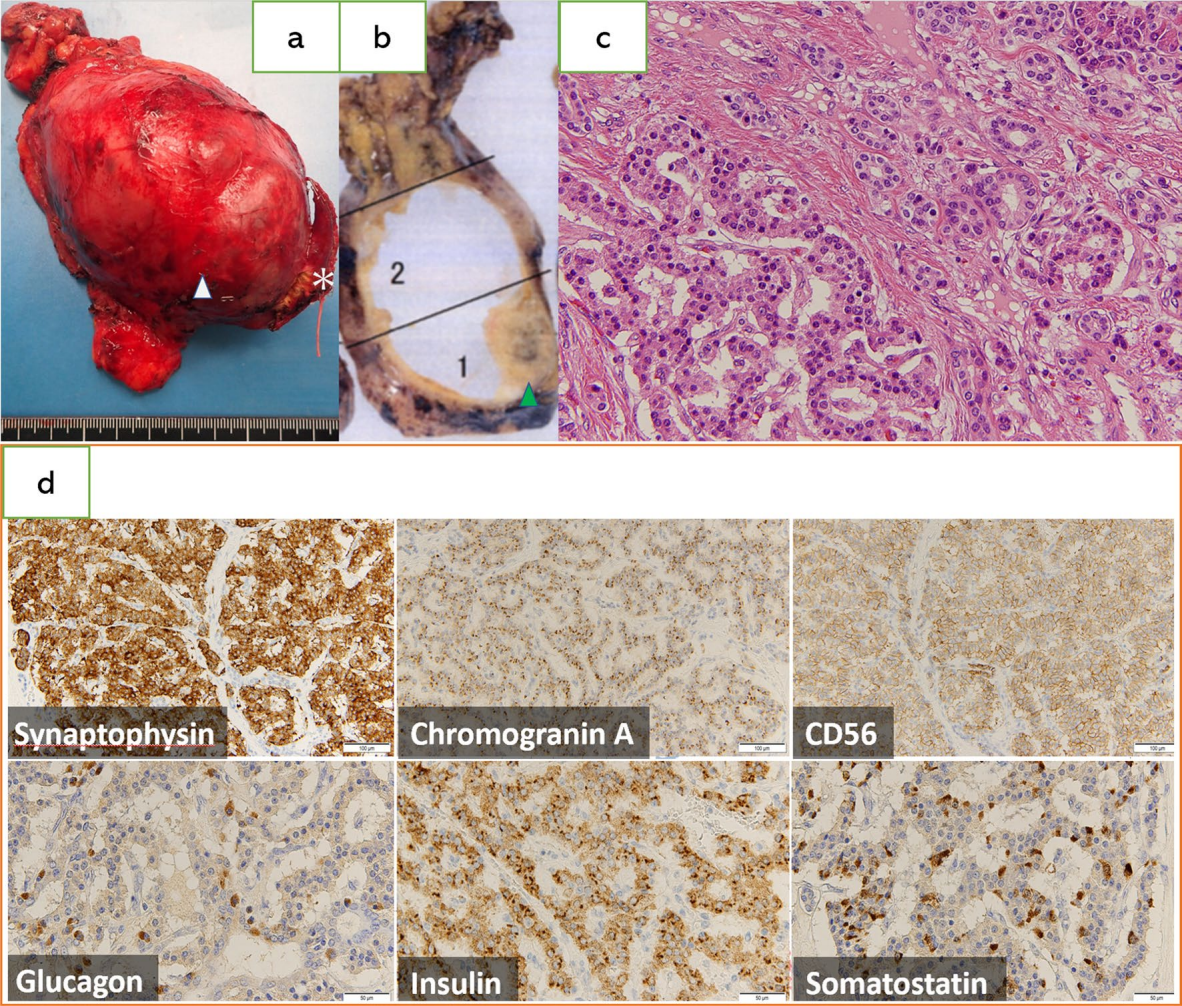
Macroscopic and microscopic findings of a neuroendocrine tumor arising in an anterior mediastinal mature teratoma. **a** The resected specimen: the arrow indicates the nodular lesion, and the asterisk indicates the partially resected lung. **b** The cut surface of the resected specimen shows a solid nodule (arrow) on part of the walls. **c** Histology findings concerning the solid nodule indicate pancreatic tissue and cord-shaped, ribbon-shaped, and cobblestone-shaped tumor cells (Hematoxylin & eosin, × 400 original magnification). **d** Immunohistochemistry analysis findings in relation to solid nodule were positive for synaptophysin, chromogranin-A, CD56, glucagon, insulin, and somatostatin

The resected tumor specimen was soft and measured 13 cm × 9 cm × 5 cm (Fig. 2a). A small portion of attached lung tissue exhibited no gross evidence of invasion. The cut surface was an encapsulated cyst. The cyst contained debris, and a solid nodule 1.5 cm in size comprised a portion of the walls (Fig. 2b). Histology findings indicated foam cells and skin appendages within the cystic space and ciliated columnar epithelium, stratified squamous epithelium, and cartilage tissue on the cyst wall. Histological examination of the solid nodule revealed pancreatic tissue, some of which exhibited cord-shaped, ribbon-shaped, and cobblestone-shaped tumor cell proliferation (Fig. 2c). On immunohistochemical analysis, the tumor cells were positive for synaptophysin, chromogranin A, CD56, glucagon, insulin, and somatostatin (Fig. 2d). Since the Ki-67 index was < 3%, the tumor was diagnosed as a mature teratoma comprising a neuroendocrine tumor, corresponding to a grade 1 pancreatic neuroendocrine tumor, according to Endocrine Tumours (4th edition). Three years postoperatively, the patient remained healthy, with no signs of recurrence.

## Discussion and conclusions

Mature teratomas are primary germ cell neoplasms derived from more than one of the three embryonic germ cell layers and comprised of fully differentiated adult tissues. They account for 5–10% of all mediastinal tumors [2]. Only 15–30% of mediastinal teratomas are malignant neoplasms, including immature teratomas, teratomas concomitant with mixed germ cell tumors, and GCTSM [3]. According to previous reports, GCTSM commonly arises from a germ cell tumor, following chemotherapy and/or radiotherapy, among patients with an initial malignant tumor; however, little is known about the incidence and clinicopathological features of GCTSM in the mediastinum owing to their rarity [4]. GCTSM have a poor prognosis because they are often diagnosed in advanced stages, with an average patient survival time of approximately nine months [1].

To date, only eight cases of neuroendocrine tumors, arising from mediastinal mature teratomas, including the present case, have been reported in the English literature [5–11]. In these eight cases, the median age (range: 24–66 years) was 37.5 years, and the median tumor diameter (range: 5–20 cm) was 8.6 cm. Three patients reported chest pain [9, 11], and one exhibited persistent dry cough [10]. In the remaining patients, the tumors were detected incidentally. All serum markers were within the normal range or not reported. None of the patients received chemotherapy or radiotherapy. Surgical resection was performed in all cases. However, in one case, incomplete resection, followed by radiotherapy, was performed [10]. In another case, the lesion infiltrated the upper lobe of the left lung and pericardium [8]. The aorta was involved in one case [10]. In our patient, the lesion had infiltrated the upper lobe of the left lung. One patient received adjuvant chemotherapy with cisplatin and bleomycin [8]. A neuroendocrine tumor and adenocarcinoma were identified in one patient [11]. Recurrence was not observed in any of the reported cases.

Carcinoid tumors comprising 40% of primary neuroendocrine tumors of the ovary, have been reported to be associated with thyroid follicle [12]; however, no associations have been reported between a neuroendocrine mediastinal tumor contained within a mature teratoma and thyroid follicle [5–11]. Tumors in our case and in another case [6] were found to be associated with pancreatic tissue, and a tumor in another case [7] were found to be associated with gastrointestinal epithelium. Actually, our case was demonstrated to be positive for pancreatic islet hormones, glucagon, insulin and somatostatin. It is well known that positive reaction for markers by immunohistochemistry and serum hormonal level and/or clinical symptom are not always correlated, and it was not expected that pancreatic elements were contained in the teratoma, we did not examined serum levels of glucagon, insulin and somatostatin. In this case, symptoms such as interstitial chest tightness and the fact that the tumor had partially infiltrated the lung may have been associated with pancreatic tissue, but this could not be proven pathologically.

No known clinical characteristics are unique to the diagnosis of mediastinal GCTSM. Mediastinal GCTSM are generally observed to be large, measuring at least 6 cm. They are typically apparent on chest radiographs [1]. On CT, GCTSM present as nodular lesions within encapsulated cystic lesions. An obtuse angle between the nodular lesion and the inner wall of the cyst, as well as extracapsular tumor growth, extending into the adjacent structures of metastasis has been reported [13]. After intravenous administration of contrast material, the nodular soft tissue is enhanced on CT [1]. While nuclear magnetic resonance imaging is not useful for diagnosing GCTSM, it is beneficial for evaluating the relationship between the mediastinum and hilar structures [14]. Some somatic-type malignancies, such as adenocarcinoma and squamous cell carcinoma in mediastinal mature teratomas, reportedly exhibit ^18^F-FDG accumulation [15]. However, there have been no reports of PET scans for neuroendocrine tumors within mediastinal mature teratomas. In our case, PET scans showed no significant ^18^F-FDG accumulation in the neuroendocrine tumor, arising from a mature mediastinal teratoma. PET scans often fail to show ^18^F-FDG accumulation in small tumors < 1 cm, even when they are malignant [16]. One study reported that the Ki-67 index correlated with FDG accumulation [17]. Therefore, in this case, the smaller neuroendocrine tumor and lower Ki-67 index may have prevented the tumor from showing FDG accumulation.

Treatment options for GCTSM have not yet been established. Surgical resection is a viable treatment option for GCTSM in cases where a tumor has not invaded, metastasized, or disseminated [1]. However, in patients suspected of developing GCTSM, surgery is indicated because damage to the tumor causes recurrence in the thoracic cavity or mediastinum [18]. Chemotherapy is also considered effective for GCTSM according to the type of somatic-type malignancy [19]. However, indications for chemotherapy have not been established. Moreover, patients with tumors that cannot be completely excised, have poorer prognoses.

Here, we report a case of a neuroendocrine tumor, arising from an anterior mediastinal mature teratoma. In this case, the tumor was excised without complications and there was no recurrence. However, a preoperative diagnosis remains challenging because neuroendocrine tumors do not exhibit ^18^F-FDG accumulation on PET. Therefore, GCTSM should be suspected in cases exhibiting CT findings of a mediastinal tumor, and surgery should be performed more carefully in such cases.

## Data Availability

All data concerning this case report are included in this published article.

## Abbreviations

CT: Computed tomography
^18^F-FDG: ^18^F-fluorodeoxyglucose
GCTSM: Germ cell tumor with somatic type malignancy
PET: Positron-emission tomography
